# Interfacial Polymerization for Colorimetric Labeling of Protein Expression in Cells

**DOI:** 10.1371/journal.pone.0115630

**Published:** 2014-12-23

**Authors:** Jacob L. Lilly, Phillip R. Sheldon, Liv J. Hoversten, Gabriela Romero, Vivek Balasubramaniam, Brad J. Berron

**Affiliations:** 1 Department of Chemical and Materials Engineering, University of Kentucky, Lexington, Kentucky, United States of America; 2 Department of Pediatrics, University of Colorado, Denver, Colorado, United States of America; Duke University Marine Laboratory, United States of America

## Abstract

Determining the location of rare proteins in cells typically requires the use of on-sample amplification. Antibody based recognition and enzymatic amplification is used to produce large amounts of visible label at the site of protein expression, but these techniques suffer from the presence of nonspecific reactivity in the biological sample and from poor spatial control over the label. Polymerization based amplification is a recently developed alternative means of creating an on-sample amplification for fluorescence applications, while not suffering from endogenous labels or loss of signal localization. This manuscript builds upon polymerization based amplification by developing a stable, archivable, and colorimetric mode of amplification termed Polymer Dye Labeling. The basic concept involves an interfacial polymer grown at the site of protein expression and subsequent staining of this polymer with an appropriate dye. The dyes Evans Blue and eosin were initially investigated for colorimetric response in a microarray setting, where both specifically stained polymer films on glass. The process was translated to the staining of protein expression in human dermal fibroblast cells, and Polymer Dye Labeling was specific to regions consistent with desired protein expression. The labeling is stable for over 200 days in ambient conditions and is also compatible with modern mounting medium.

## Introduction

The determination of spatial patterns of protein expression in biological samples is a cornerstone of modern clinical diagnostic and biological research. Protein identification and localization is typically achieved through incubation of the sample with labeled antibodies against the protein of interest. While direct labeling of the target antibody is sufficient for localization of abundant proteins in fluorescent imaging, amplification of the signal is typically required to label proteins for brightfield observation of samples where dilute proteins can be difficult to observe colorimetrically. Horseradish peroxidase (HRP) amplification is a common method for amplifying the label of a poorly expressed protein in cells and tissues. The basic concept uses the incubation of HRP enzyme coupled to antibody location, typically through biotinylated antibodies and HRP-avidin conjugates [1]. The specificity of the antibody binds the enzyme to regions expressing the protein of interest. When the sample is subsequently immersed in a solution of hydrogen peroxide and diaminobenzidine, the HRP rapidly converts the diaminobenzidine to yield an insoluble brown product. Under ideal conditions, the presence of the brown product is isolated to regions of expression of the target protein. Unfortunately, nonspecific HRP signal is common from endogenous peroxidases naturally residing in the tissue [2]. The presence of these active enzymes in the sample tissue requires additional sample processing to quench their activity [3]. Incomplete quenching can lead to false positives or inconclusive staining. Further, fine localization of HRP staining is an empirical process, where over-amplification commonly results in significant diffusion of the signal away from the targeted protein expression.

**Figure 1:**
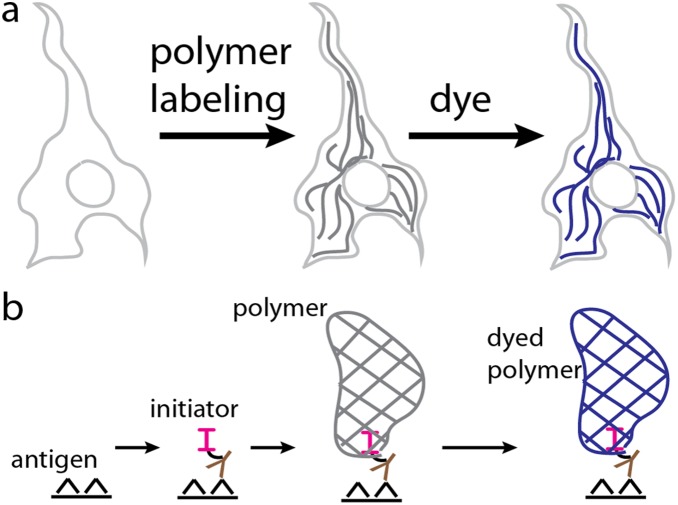
Polymer Dye Labeling concept at the (a) cellular level and (b) molecular level. A polymerization initiator is localized to site of antigen through antibody and biotin-streptavidin labeling. Interfacial hydrogel polymerization occurs only at regions labeled with initiator. The hydrogel is colorimetrically labeled through an affinity dye.

Polymerization based amplification (PBA) recently emerged as a signal amplification approach which does not suffer from diffusional loss of localization or endogenous signal [4], [5]. PBA uses interfacial polymerization as the basis for depositing a large amount of label at the site of a biological recognition event (e.g. antibody/antigen) [6]–[9]. Both the presence of a polymerization initiator and reactive monomers are required for the formation of polymer. The PBA approach couples the spatial localization of the polymerization initiator to that of a specific protein recognition event (Fig. 1). Wherever the antibody recognizes the target protein, a polymerization initiator is immobilized. Upon addition of monomer and the appropriate excitation energy, a polymer coating is formed through the deposition of many monomers at the site of an initiation event. The process has been previously demonstrated on microarrays to specifically form polymer films from as few as 3 binding events per square micron allowing great sensitivity and specificity at antibody concentrations that will limit non-specific background staining [10].

PBA has limitations with respect to sample archival. On cells, PBA has exclusively utilized fluorescent visualization of polymerization events [10], [11]. While PBA has shown strong stability of fluorescent signal during standard imaging conditions, a colorimetric stain would be advantageous for long-term sample storage and archiving. Additionally, the fluorophores currently used in PBA are typically quenched upon addition of mounting medium utilized for long term storage (S1 Fig.) [11]. This challenge would also be overcome by a non-fluorescent, colorimetric amplification strategy.

Here, we seek to adapt PBA to serve as a colorimetric, signal-amplification scheme. Our general approach, termed Polymer Dye Labeling involves the specific loading of the interfacial polymer with dyes. The interfacial polymer typically used in PBA is Polyethylene glycol diacrylate (PEG diacrylate), which has been demonstrated to have specific staining with common dyes [8], [12]–[14]. Literature shows both Evans Blue [14] and eosin [9] to be effective in staining PEG diacrylate polymers. In particular, the Sikes group has established the use of eosin stained microarrays for colorimetric assays of oligonucleotide and protein expression, with strong signal to noise [8], [9], [15]. Critically, the ability of eosin to non-specifically stain many cellular components present in biological samples [16] precludes its use in Polymer Dye Labeling to detect specific targeted cellular substrates. We seek to develop a dye system of comparable staining intensity to the eosin dye, but with reduced affinity for common cellular components in biological cells. In all, Polymer Dye Labeling is expected to draw from the advantages of PBA (large signal, excellent localization, and specificity of action) while adding colorimetric capability to allow improved sample archiving.

Our evaluation of Polymer Dye Labeling builds on prior PBA technology [4], [6], [7], [10]–[12], [17]–[21]. We first confirm the expected process of PBA through quantifying the deposition of initiator and polymer on control glass surfaces. We then examine the loading of eosin and Evans Blue dyes into these interfacial coatings through quantification of color change. We then extend this work to the labeling of cells by Polymer Dye Labeling. On a culture of human dermal fibroblast samples, we confirm the stability of the Polymer Dye Labeling signal over 200 days, and also demonstrate the compatibility of the Polymer Dye Labeling technology with conventional mounting media used in sample archiving.

## Materials and Methods

### Materials

Epoxy functionalized slides were purchased from CEL Associates. Biotinylated bovine serum albumin (bio-BSA), streptavidin, eosin-isothiocyanate, eosin-y, Monoclonal mouse IgG1 anti-vimentin (V9; catalogue #V6389), 10× phosphate-buffered saline (PBS), Triton-X 100, trypsin, PEG diacrylate (Mn = 575), triethanol amine, and 1-vinyl-2-pyrrolidinone were purchased from Sigma Aldrich (St Louis, MO). Monoclonal mouse IgG1 anti-NPC (MAb414) was purchased from Covance (Princeton, NJ; catalogue #MMS-120P). Biotinylated polyclonal goat IgG anti-mouse IgG (H+L; catalogue #BA-9200) and Vectashield hardset mounting medium was purchased from Vector Laboratories (Burlingame, CA). Bovine serum albumin (BSA), methanol, and ethanol (absolute) were purchased from Fisher Scientific (Waltham, MA). Paraformaldehyde was purchased from Electron Microscopy Sciences (Hatfield, PA). Streptavidin-Alexa488 conjugates were purchased from Life Technologies (Grand Island, NY).

RPMI-1640 cell culture media was purchased from Cellgro and supplemented with 10% fetal bovine serum (FBS, Gibco, Carlsbad, CA), 100 U/mL Penicillin, 10 mg/mL Streptomycin (Gibco) prior to use. Normal human dermal fibroblasts (#CC-2511) were purchased from Lonza (Basel, Switzerland).

Streptavidin-eosin (SA-initiator) was prepared as described previously [6]. PBSA was prepared by adding 1 mg/mL bovine serum albumin 1x PBS. Monomer mix was prepared immediately prior to use and consists of 25 wt% PEG diacrylate, 21 mM triethanol amine, 35 mM 1-vinyl-2-pyrrolidinone, and 0.05 wt% Nile red fluorescent nanoparticles in deionized water.

### Biotin microarray printing

Epoxy functionalized glass slides were rinsed with ethanol, dried under a stream of nitrogen, and placed on the stage of the Affymetrix (Santa Clara, CA) GMS 417 Arrayer. BSA solutions were prepared keeping a constant 1 mg/mL concentration of BSA in PBS, and varying the fraction of BSA that is biotinylated. Twelve solutions were prepared at the following concentrations of biotinylated BSA: 1 mg/mL, 400 µg/mL, 160 µg/mL, 64 µg/mL, 26 µg/mL, 10 µg/mL, 4 µg/mL, 1.6 µg/mL, 650 ng/mL, 260 ng/mL, 100 ng/mL, 0 ng/mL. Arrays consisted of 24 spots, where each solution was duplicated on each array, and four identical arrays were printed on each glass slide. Only the two centermost arrays were used, as the polymerization light source can only irradiate two arrays at a time. Slides were printed under 60% relative humidity in a single batch of 25 slides.

### Microarray polymerization, staining, and imaging

Slides were blocked in PBSA for 10 minutes, incubated in 1.0 µg/mL SA-initiator in PBSA for 20 minutes, and rinsed with PBSA. These initiator-labeled arrays were then scanned in an Affymetrix Microarray Scanner (Model 428) using 532 nm laser excitation and a 551±7 nm band pass emission filter. Files were exported to ImageJ for analysis of array spot intensity. Fluorescent data are reported as the mean and standard deviation of 16 measurements (two duplicates spots per array, two arrays per slide, four independent preparations of a single slide).

Slides were then immediately placed in a Chip Clip (Whatman, Little Chalfont, UK) with a two well FAST slide (Whatman) with 400 µL of monomer mix in each well. Samples were purged with humidified nitrogen in a clear plastic bag for 5 minutes. Then, the slide was irradiated for 20 minutes with collimated, 10 mW/cm^2^, 530 nm LED light (Thorlabs, Newton NJ, model M530L3) under a constant stream of humidified nitrogen. After irradiation, samples were rinsed with water. The samples were then incubated in a 1 mg/mL solution of the indicated dye for 20 minutes. Evans Blue was prepared in PBS, while eosin was prepared in an aqueous solution of 50% methanol to promote solubility. Slides were dried under a stream of nitrogen, and imaged using an Epson Perfection 4490 Photo flat-bed document scanner at a resolution of 2400 dpi. Only the two arrays most-centered under the LED irradiation were analyzed owing to radial non-uniformities in the irradiation intensity. Greyscale values of the fractional darkness of each spot were collected for each spot using ImageJ. Fractional darkness is defined as 1.00 minus the fractional greyscale value of spot brightness. The thickness of each polymer spot was measured with a Dektak 6 M stylus profilometer.

Limit of detection is defined as the lowest concentration of biotinylated BSA of a different mean when compared to the lower concentrations with at least 95% confidence by student t-test. The saturation range is defined as the high concentration range of biotinylated-BSA where the mean measurement is not different from each other with at least 95% confidence by a student t-test. The dynamic range is defined as the concentration range between the limit of detection to the saturation region.

### Immunolabeling of cells

Dermal fibroblasts were cultured on 8 well chamber slides in media at 37 C in 5% CO_2_ until ∼80% confluent. The cells were rinsed with cold PBS, and fixed in 4% paraformaldehyde in PBS for 10 minutes. Fixed cells on chamber slides were stored in PBS at 4°C for up to 30 days prior to use with no observed change in staining intensity. Cells were permeablized with 0.1% Triton X-100 in PBS for 5 minutes and blocked with PBSA for 10 minutes. Then, slides were incubated in the appropriate primary antibody at the appropriate dilution in PBSA (anti-NPC at 1∶1,000 or anti-vimentin at a 1∶5,000) for 40 minutes and rinsed with PBSA. The cells were contacted with biotinylated antibodies against mouse IgG at 1∶400 dilution in PBSA for 4 minutes and rinsed with PBSA. These samples were then ready for either Polymer Dye Labeling or control labeling with Alexa488.

For Polymer Dye Labeling, the cells were incubated in a 25 µg/mL solution of SA-initiator in PBSA for 20 minutes and rinsed with PBS. 80 µL of monomer mix was added to each well, and the slides were polymerized for 20 minutes with collimated, 10 mW/cm^2^, 530 nm LED light (Thorlabs model M530L3) under a constant stream of humidified nitrogen. After irradiation, samples were rinsed with water, and incubated in a 1 mg/mL solution of Evans Blue dye in PBS for 20 minutes. Samples were briefly rinsed with PBS, and then imaged on a Nikon (Tokyo, Japan) Ti-U inverted microscope using a 60x oil immersion objective with a Nikon DS-Ri1 12 MP cooled color CCD camera.

For control experiments, cells labeled with biotinylated secondary antibodies were contacted with 1 µg/mL streptavidin-Alexa488 in PBSA for 20 minutes and were immediately imaged on a Nikon Ti-U inverted microscope as before except with epifluorescent imaging in the FITC channel.

Greyscale values of the fractional darkness of each spot were collected for each spot using ImageJ. Fractional darkness is defined as 1.00 minus the fractional greyscale value of spot brightness. Background (non-cell region) darkness was subtracted from both the signal (nucleus region) and noise (cytoplasm region). Signal to noise is defined by the division of the signal value by the noise value.

## Results and Discussion

Our goal is to develop a colorimetric alternative to enzymatic amplification which is not hampered by non-specific amplification by endogenous enzymes or through diffusional loss of signal localization. Our approach, “Polymer Dye Labeling,” is a multi-step process where 1) polymerization initiator is localized to the site of antigen expression, 2) an interfacial polymer coating is grown from the surface-grafted initiator, and 3) dye is loaded into the polymer. Our approach is to first study the fundamental relationship between initiator binding and the intensity of Polymer Dye Labeling. Then, we investigate the comprehensive Polymer Dye Labeling process when applied to the labeling of protein expression in cultured human dermal fibroblasts.

### Characterization of recognition, polymerization, and dye association

**Figure 2:**
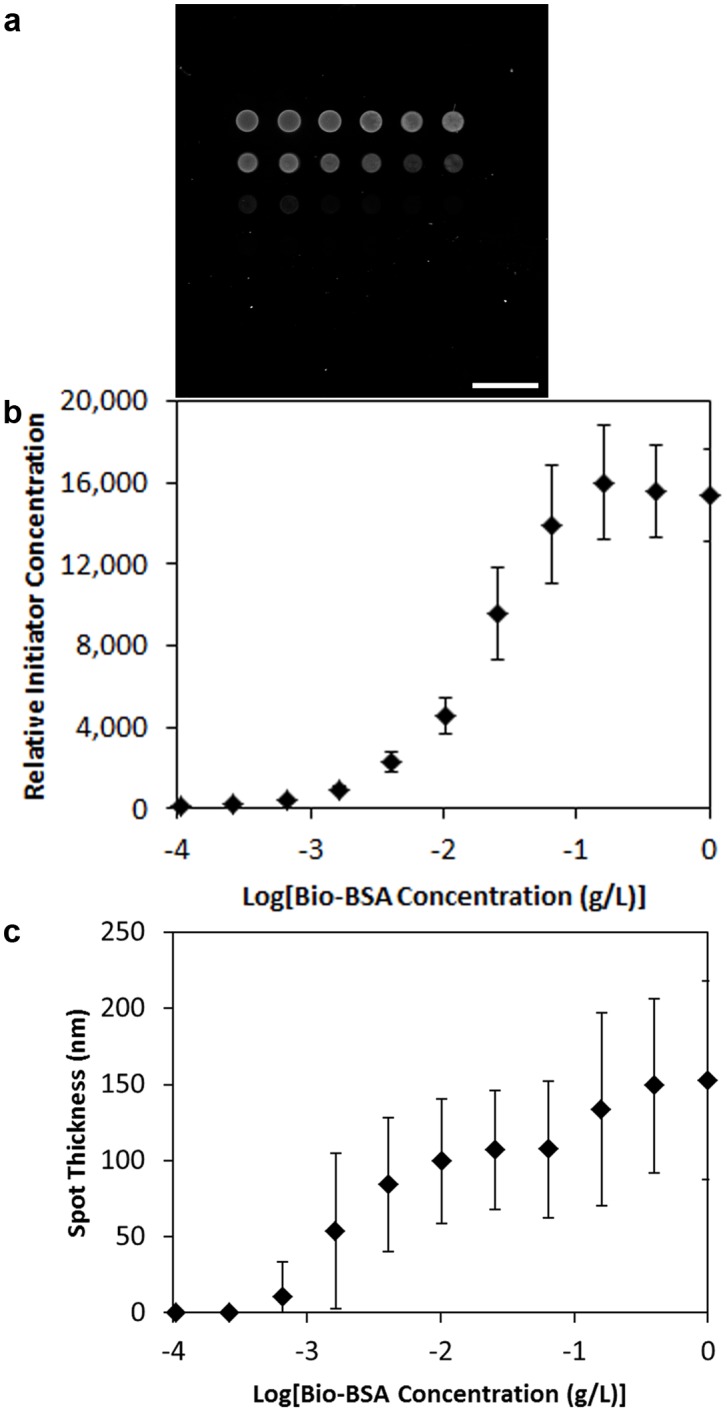
Imaging of initiator concentration for microarray. a) Fluorescent microarray scanner measuring relative abundance of initiator prior to polymerization labeling. Scale bar = 1 mm. b) Relative initiator concentration on surface for spots printed from the indicated concentration of biotinylated-BSA and reacted with the SA-initiator complex. Measurements based on initiator fluorescence (ex/em = 525/545 nm). c) Thickness of spots of indicated Bio-BSA concentration. Data are mean ± standard deviation. n = 16.

Bio-BSA was printed into microarrays on an epoxy coated slide, and blocked with PBSA. Recognition of the SA-initiator with the biotin of the bio-BSA, was quantified through measuring the fluorescence of the eosin initiator in biotin-expressing regions. A solution of the SA-initiator conjugate at 1 µg/mL in PBSA was contacted with each microarray for 20 minutes, and excess conjugate was rinsed briefly with PBSA prior to capturing a fluorescent image with a microarray scanner (Fig. 2a). The fluorescence of each spot was measured using ImageJ, and plotted against the corresponding concentration of Bio-BSA in the printing solution (Fig. 2b). The relative initiator concentration is indistinguishable from the background at printed solution concentrations less than 10^−3^ g/L of Bio-BSA (limit of detection, p = 10^−14^). There is a two log fluorescent dynamic range, and saturation above 10^−1^ g/L of Bio-BSA (p = .043). Initiator binding is restricted to printed regions, and printed spots containing only BSA did not exhibit fluorescence greater than that of non-printed regions. The specificity of binding in this study is consistent with previous reports of initiator binding based on antibody-antigen [10], [11], [20] or Streptavidin-biotin [6], [17] interactions.

Interfacial polymerization is accomplished through the immersion of an initiator-primed surface in a PEG diacrylate monomer solution. Polymerization proceeded with a 20 minute exposure to 10 mW/cm^2^, 530 nm irradiation under a nitrogen atmosphere. The resulting arrays of polymer spots were measured by profilometry to determine the sensitivity and magnitude of the polymerization reaction (Fig. 2c). As expected, polymer growth was restricted to regions of initiator-labeling, supporting the specificity of the polymerization process. The limit of detection was identical to that of the fluorescence arrays (10^−3^ g/L of Bio-BSA, p = .001). The dynamic range of polymer thickness extended to 10^−1^ g/L of Bio-BSA (p = .01), and was identical to that of the dynamic range of initiator concentration on the surface, supporting prior reports of the polymerization reaction being limited by the initiator concentration [8].

**Figure 3:**
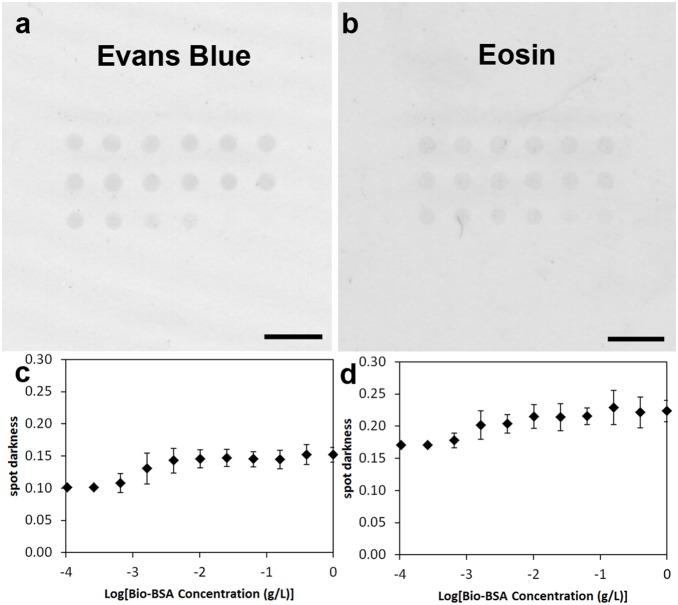
Colorimetric imaging of Polymer Dye Labeling. a) Greyscale image from optical document scanner after Polymer Dye Labeling with Evans Blue dye. b) Greyscale image from optical document scanner after Polymer Dye Labeling with eosin dye. Scale bars = 1 mm. c) darkness of Evans Blue dyed spots of indicated Bio-BSA concentration. d) Darkness of eosin dyed spots of indicated Bio-BSA concentration. Data in c) and d) are mean ± standard deviation. n = 12.

Incubation of the PEG diacrylate hydrogels in a dye is expected to alter the color of the polymer. We are investigating Evans Blue as a candidate dye for strong specific staining of the polymer with minimal nonspecific staining of cellular material. Arrays of PEG diacrylate polymer films were incubated in 1 mg/mL Evans Blue for 20 minutes, and upon removal, the polymer spots were darkened, while the surrounding glass slide remained unstained (Fig. 3a). The darkness of the spots was quantified and plotted against the printed concentration of bio-BSA (Fig. 3c). Again, the limit of detection was identical to that of the polymer thickness and the initiator concentration (10^−3^ g/L of Bio-BSA, p = .0004). Critically, the dynamic range of the staining was negligible, and saturation range began at the next data point (4×10^−3^ g/L of Bio-BSA, p = .008). As a result, the colorimetric response was largely binary. When compared to the use of 1 mg/mL eosin as the polymer dye (Fig. 3b, 3d), Evans Blue has a greater magnitude of colorimetric labeling (p = 10^−63^) of the polymer stained regions but different levels of background staining (p = 10^−5^). The limit of detection (10^−3^ g/L of Bio-BSA, p = .0008) and beginning of the saturation range (10^−2^ g/L of Bio-BSA, p = .0008) for eosin are similar to the Evans Blue labeling. This indicates Evans Blue is a potential alternative to eosin in colorimetric staining of PEG diacrylate hydrogels in microarray settings. The use of eosin dyes on hydrogel microarrays has already demonstrated effectiveness in a colorimetric detection of biological species [8], and the use of a blue dye may improve ease of use over the pink color associated with eosin-dyed hydrogels.

**Figure 4:**
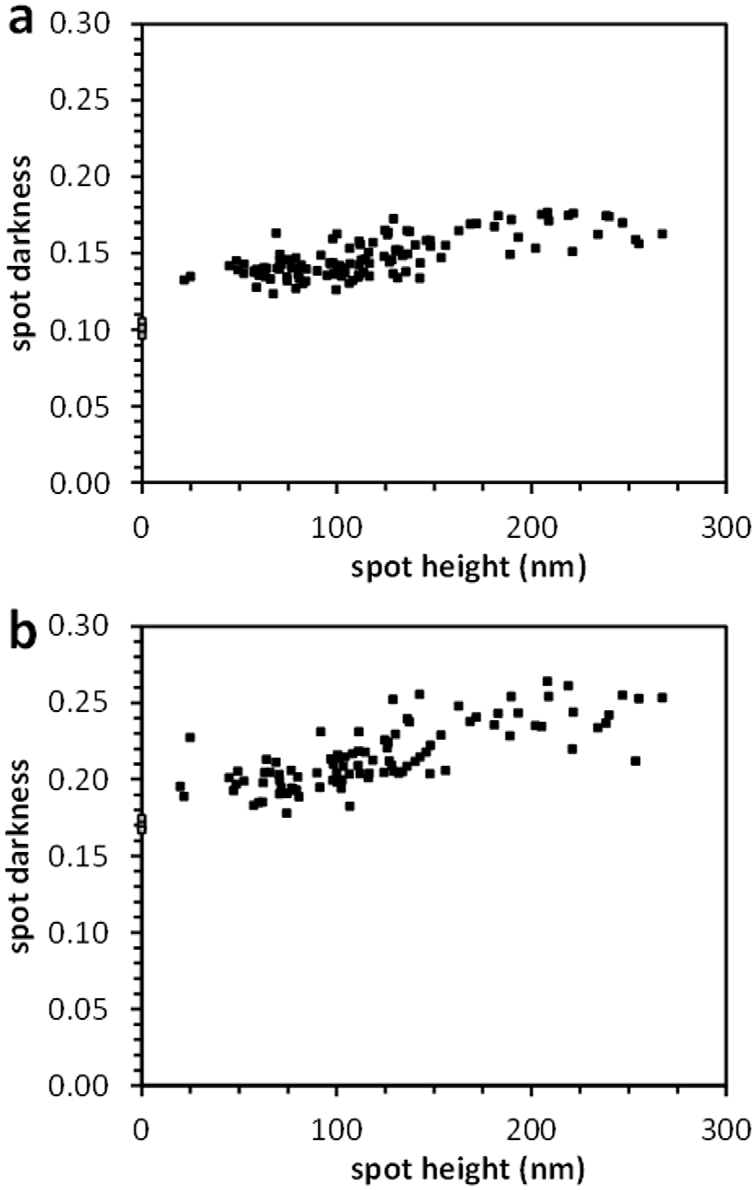
Relationship between polymer spot thickness and spot darkness after Polymer Dye Labeling. a) Polymer Dye Labeling with Evans Blue dye. b) Polymer Dye Labeling with eosin dye. Each data set includes at least 6 microarrays from 3 independent experiments. Black squares indicate array data. Grey squares indicate mean value of non-specific regions for each experiment.

To directly compare the effectiveness of the dye-labeling step, we related the darkness of each spot to the thickness of the hydrogel at that location, providing a relationship of how much dye is absorbed per unit thickness by the PEG diacrylate hydrogels. Applying a linear relationship (slope  = 1.78×10^−4^ darkness units per nm) to the Evans Blue data is consistent with the expected increase in spot darkness with a longer path length through the dyed polymer (Fig. 4a), yet this fit is statistically different than the data (p = .01), indicating a poor fit. A linear relationship (2.7×10^−4^ darkness units per nm) is observed for the eosin-dyed polymer spots (Fig. 4b), without a statistical difference between the data and the linear fit (p = 0.06). Additionally, the eosin associated with the initiator is not perceptible through visual observation prior to immersion of the hydrogel in eosin. All darkness of the spot is attributed to the post-polymerization dying. While the magnitude of the spot darkness is higher for the eosin dyed spots than the Evans Blue dyed spots, there is a comparable difference in nonspecific darkness on the glass slide. Here, we show the eosin labeling of the polymer is specific. In previous studies, greater signal to noise has been reported by others through the use of 20-fold higher concentrations of eosin [9]. Higher concentrations of eosin or Evans Blue were not used in the present study, in an effort to limit nonspecific staining in subsequent cell studies.

The diameter of the dye labeled polymer spots was determined by optical microscopy to be 340±20 µm (Fig. 3a, 3b), and this value was within measurement error of the spot size of the original initiator labeled arrays of 350±20 µm (Fig. 2a). The lack of detectible polymer overgrowth is promising for the localization of the polymer to the site of protein expression.

### Labeling of protein expression in cells

**Figure 5:**
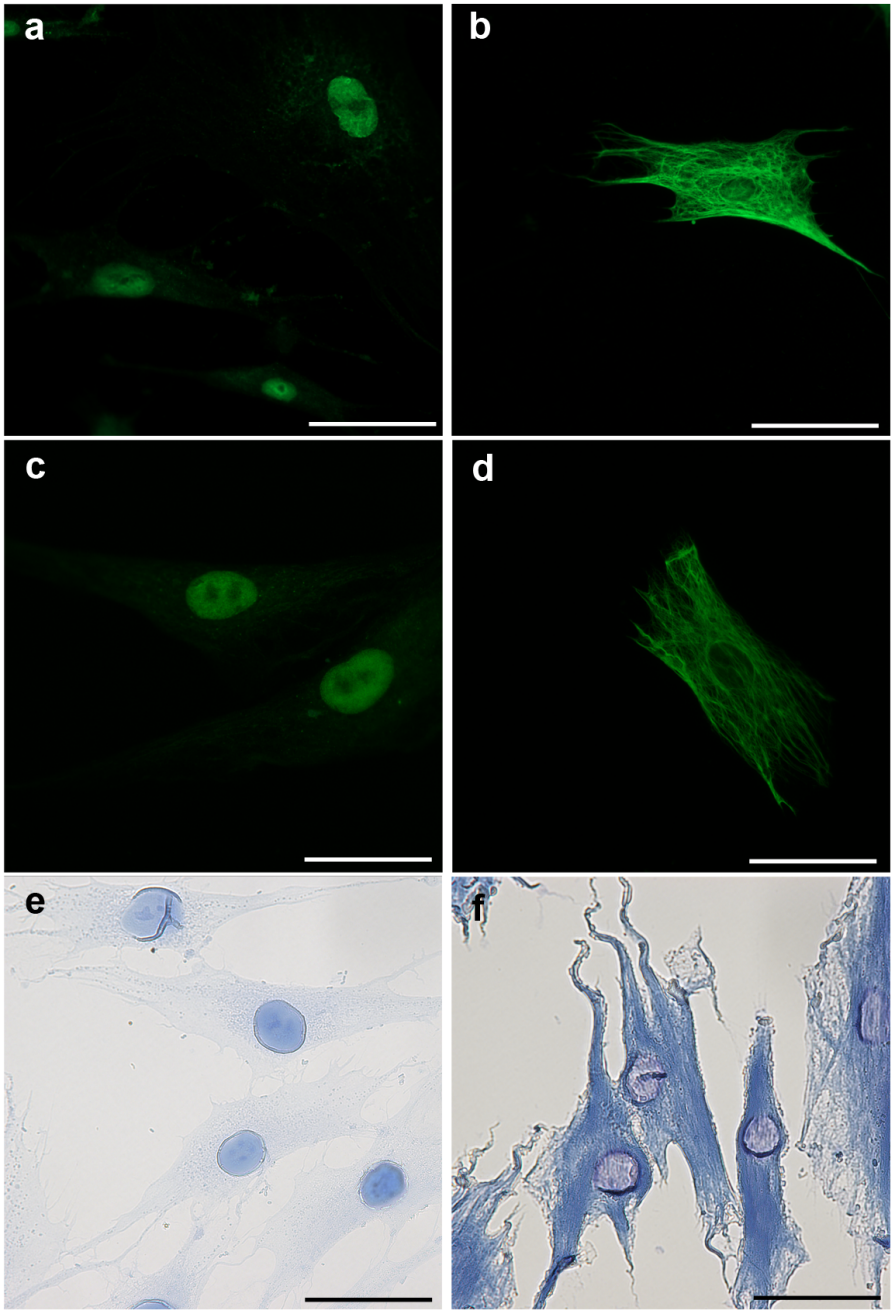
Comparison of Polymer Dye Labeling with immunofluorescent labeling in human dermal fibroblasts. Control fluorescent staining of nuclear pore complex (a) and vimentin (b) using Streptavidin-Alexa488. Initiator localization when using antibodies against nuclear pore complex (c) and vimentin (d). Dyed Polymer localization when using antibodies against nuclear pore complex (e) and vimentin (f). Scale bars are 50 µm.

The transition from a controlled microarray environment to a biological substrate introduces additional challenges to label specificity. Every step in the amplification process must be specific to the region of antibody/antigen recognition. For the localization of the initiator, the specificity is dictated by specific binding of the antibodies and the SA-initiator complex [4]. When antibodies against nuclear pore complex (NPC) are used on a fixed, permeabilized, and blocked dermal fibroblast, the initiator fluorescence is isolated to the nuclear membrane (Fig. 5c). When the NPC primary antibodies are replaced with antibodies against vimentin, the initiator fluorescence is localized to vimentin, a fibrous structural component which stretches across the cytoplasm (Fig. 5d). Control experiments using standard streptavidin-Alexa488 instead of the SA-initiator show identical patterns of expression (Fig. 5a, 5b), supporting the appropriate protein specificity of the initiator localization. The signal intensity from labeling with SA-initiator (signal to noise 4.53±0.36) and streptavidin-Alexa488 (signal to noise 4.23±0.80) are fully described in S2 Table. These findings are consistent with prior work in polymerization amplification [6], [11].

Upon addition of the PEG diacrylate monomer mix to the initiator-labeled cells and irradiation with 10 mW/cm^2^, 530 nm (green) light, an interfacial polymer is formed on only surfaces expressing the target protein. Unreacted monomer is rinsed away with PBS, and the polymer-labeled cells are immersed in 1 mg/mL Evans Blue in PBS. While both eosin and Evans Blue are capable of specific staining in a microarray setting, the non-specific staining of eosin for cytoplasmic proteins and collagen precludes its use for Polymer Dye Labeling on most biological substrates [16]. As such, only Evans Blue was used in the cell staining studies. In the case of NPC labeled cells, the blue staining of the Polymer Dye Labeling (Fig. 5e) is consistent with the fluorescent control NPC staining, where the nuclear membrane is labeled. This nuclear staining is significantly darker than any nonspecific staining of non-polymer labeled fibroblasts (p = 3×10^−8^, S2 Fig.). Similarly, the Polymer Dye Labeling of vimentin is specific to these cytoskeletal components, with appropriate alignment of fibers towards cellular extensions (Fig. 5f). Taken together, the cellular labeling studies are supportive of the specificity of Polymer Dye Labeling in biological environments. Further, the intensity of staining is consistent with the expected amplification resulting from the reaction of many monomers at the site of initiation.

In the context of biological research, colorimetric staining allows independence from fluorescent analysis and associated costs. Colorimetric staining is almost exclusively accomplished with enzymatic amplification of the label and enzymatic labeling has the fundamental challenge of nonspecific labeling from endogenous enzymes and diffusion. Importantly, our work was performed in the absence of any additional steps to quench endogenous enzyme activity, as the routes for nonspecific polymerization initiation are currently undetected. A limitation of the current embodiment of polymer dye labeling is the need for a photopolymerization light source. The light source utilized here (Thorlabs LED, <$1000 US) is significantly less expensive than a fluorescent microscope which requires additional filters and optics. Further, other modes of polymerization based amplification are based on non-light driven polymerizations [5], [12], [21]–[25]. The future incorporation of ATRP or other modes of polymerization would further reduce the capital cost of polymer dye labeling.

### Suitability for Sample Archiving

**Figure 6:**
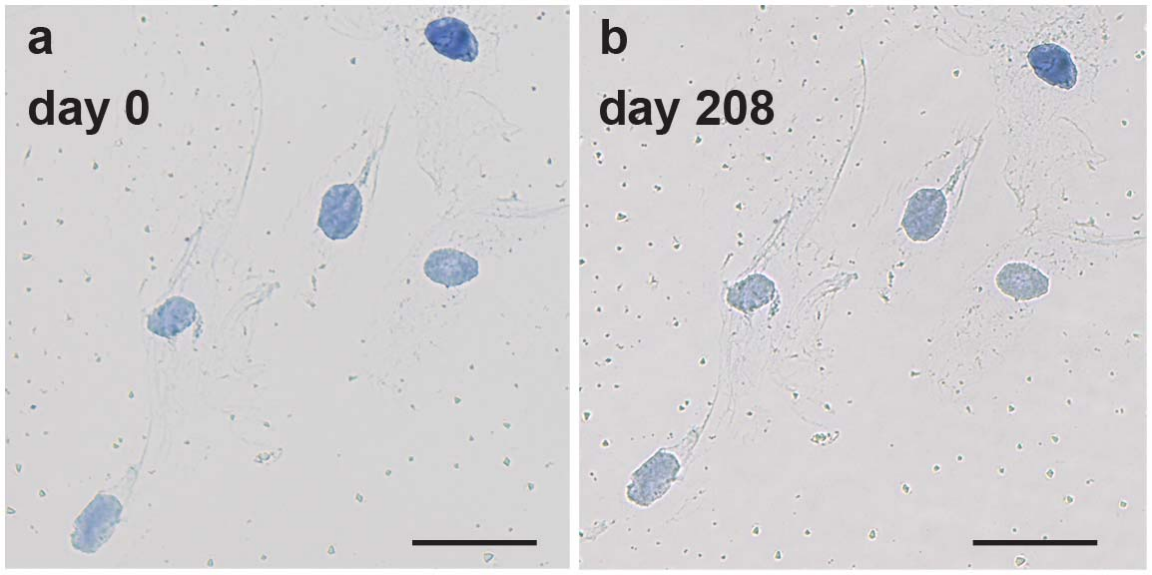
Labeling Stability of Polymer Dye Labeling. Polymer Dye Labeling of nuclear pore complex immediately after staining (a) and 208 days after staining (b). Scale bars are 50 µm.

Signal stability is a major advantage of a colorimetric staining over a fluorescent approach. We challenged the stability of cells polymer dye labeled cells with storage at ambient conditions. Specifically, the samples were imaged immediately after Polymer Dye Labeling for nuclear pore complex and again after being stored in a drawer for 208 days (Fig. 6). The darkness of the nucleus when stained (0.363±0.088) is comparable to the darkness of the nucleus 208 days after the staining (0.343±0.091). The only observable differences between the images were a slight reorientation of the frame and an increase in small optical aberrations attributed to environmental contaminants (dust, bacteria, etc.). The storage had no significant impact on the intensity or localization of staining, indicating promise for the application of Polymer Dye Labeling to long term sample archiving.

We also evaluated the stability of Polymer Dye Labeling signal when using a mounting medium. Prior studies using fluorescent PBA to label proteins has been exclusively executed in the absence of mounting medium, as the fluorescence is completely quenched in the presence of mounting media (S1 Fig.) [11]. This is a significant limitation, as mounting medium is commonly integrated into conventional imaging and archiving protocols to improve image quality and to preserve signal.

**Figure 7:**
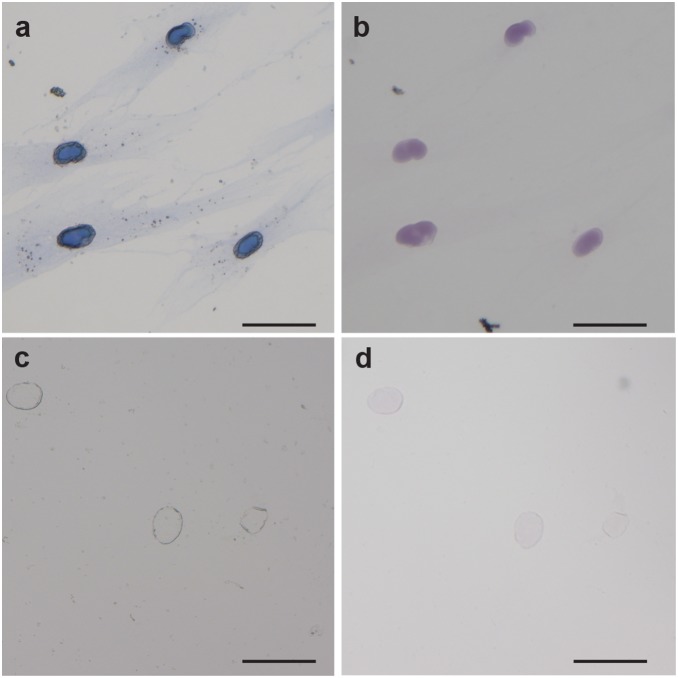
Compatibility of Polymer Dye Labeling with Vectashield mounting medium. Polymer Dye Labeling of nuclear pore complex imaged (a) dry or (b) in Vectashield hardset mounting medium. Polymer coated nuclei without Evans Blue dye imaged (c) dry or (d) in Vectashield hardset mounting medium. Scale bars are 50 µm.

NPC expression was stained through four variants of Polymer Dye Labeling: dry with Evans Blue, mounted with Evans Blue, dry without Evans Blue, and mounted without Evans Blue. Images are presented in Fig. 7, while the darkness of the stain in these images was measured with ImageJ and compiled in Table 1. For dry imaging of Polymer Dye Labeling, a blue nucleus is clearly observed (signal/noise ∼7) in contrast to minimal nonspecific signal in the cytoplasm (Fig. 7a). Vectashield hardset mounting medium was added to the sample according to manufacturer’s instructions, coverslipped and imaged (Fig. 7b). While the overall darkness of the stain decreased, the signal/noise almost tripled that of the dry Polymer Dye Labeling. This is attributed to a large decrease in the nonspecific staining of the cytoplasm.

**Table 1:** Staining intensity for Polymer Dye Labeling of nuclear pore complex.

Sample	Signal^a^^,^ c		Noise^b^^,^ c		Signal/Noise	
	Mean	Standard Deviation	Mean	Standard Deviation	Mean	Standard Deviation
Polymer Dye Labeling dry	0.333	0.009	0.049	0.004	6.9	0.7
Polymer Dye Labeling Mounted	0.191	0.016	0.010	0.003	20.9	8.2
Polymer Dry	−0.001	0.001	−0.004	0.002	0.2	0.6
Polymer Mounted	0.016	0.006	0.005	0.014	1.2	2.3

a -Signal is defined as the darkness of the nucleus.

b -Noise is defined as the darkness of the cytoplasm.

c -Values are relative increase over empty region of slide.

The most striking change with sample mounting was the change in color of the Polymer Dye Labeling from blue to violet. To verify this different color of labeling is attributed to the use of the Evans Blue dye, we polymerized in response to NPC with the omission of the Evans Blue dye (Fig. 7c). This dry, undyed sample shows negligible signal yet did impart some contrast in the image, owing to the change in refractive index between the polymerized nucleus and the background. Upon addition of mounting medium to this sample, a slight violet tint is imparted on the interfacial polymer covering the nucleus (Fig. 7d). The magnitude of the mounting medium’s contribution to the signal is low (signal/noise ∼1), supporting the Evans Blue dye as the dominant mechanism for staining. As the dark violet color of the polymer is only observed when both Evans Blue and mounting medium are used, it is likely the change in the chemical environment of the dye is altering the absorption characteristics. Similar shifts in absorption peak position are commonly observed in many light-absorbing molecules (photoinitiators [26], fluorophores [27], [28], etc.) with a change in solvent.

While enzymatic amplification methods are also stable over prolonged times and are compatible with modern sample archiving methods, polymerization based methods have greater site-specificity than enzymatic amplification [11]. The present findings clearly address the prior limitations in archiving of polymerization amplification samples, delivering a plausible path forward for a new colorimetric technique with all of the positive attributes of both enzymatic and polymerization techniques.

## Conclusions

Polymer Dye Labeling is based on interfacial polymerization which is specific to the site of the targeted protein, and these target-specific polymer coatings are then stained with Evans Blue dye. As a result, a dye-loaded polymer is isolated to regions of protein expression. In microarray studies, the use of Evans Blue provides a comparable contrast to an unstained background as eosin dyes. Application of Polymer Dye Labeling to immunostaining of cultured cells allowed bright field observation of both the spatial protein expression and cell morphology. The labeling of protein expression is stable over several months. Prior polymerization labeling approaches were incompatible with mounting medium, but Polymer Dye Labeling maintains signal intensity and localization in common mounting media. We conclude that Polymer Dye Labeling will allow colorimetric visualization of the spatial localization of targets within a cell to leverage the highly sensitive and specific aspects of Polymerization Based Amplification.

## Supporting Information

S1 Fig **Quenching of fluorescent PBA by mounting medium.** Human dermal fibroblasts were cultured on 8-well chamber slides, fixed, permeablized, blocked, labeled against nuclear pore complex, and polymerized in the presence of nile red fluorescent nanoparticles. The same representative frame imaged in brightfield (a) and in epifluorescent mode (c). After mounting with Vectashield hardset mounting medium, the same location was imaged in brightfield (b) and epifluorescent (d) imaging modes. Scale bars are 80 µm.
(TIF)

S2 Fig **Control study showing limited nonspecific labeling of cells.** Human dermal fibroblasts were cultured on 8-well chamber slides, fixed, permeabilized, blocked, and incubated in Evans Blue dye (1 mg/mL in PBS).
(TIF)

S1 Table **Temporal staining intensity for Polymer Dye Labeling of nuclear pore complex.**
(DOCX)

S2 Table **Staining intensity for immunofluorescent labeling of nuclear pore complex.**
(DOCX)
