# Supplementary figures and images for: Interfacial Polymerization for Colorimetric Labeling of Protein Expression in Cells
Source: PLoS One. 2014 Dec 23;9(12):e115630. doi: 10.1371/journal.pone.0115630 (PMC4275217; doi:10.1371/journal.pone.0115630)

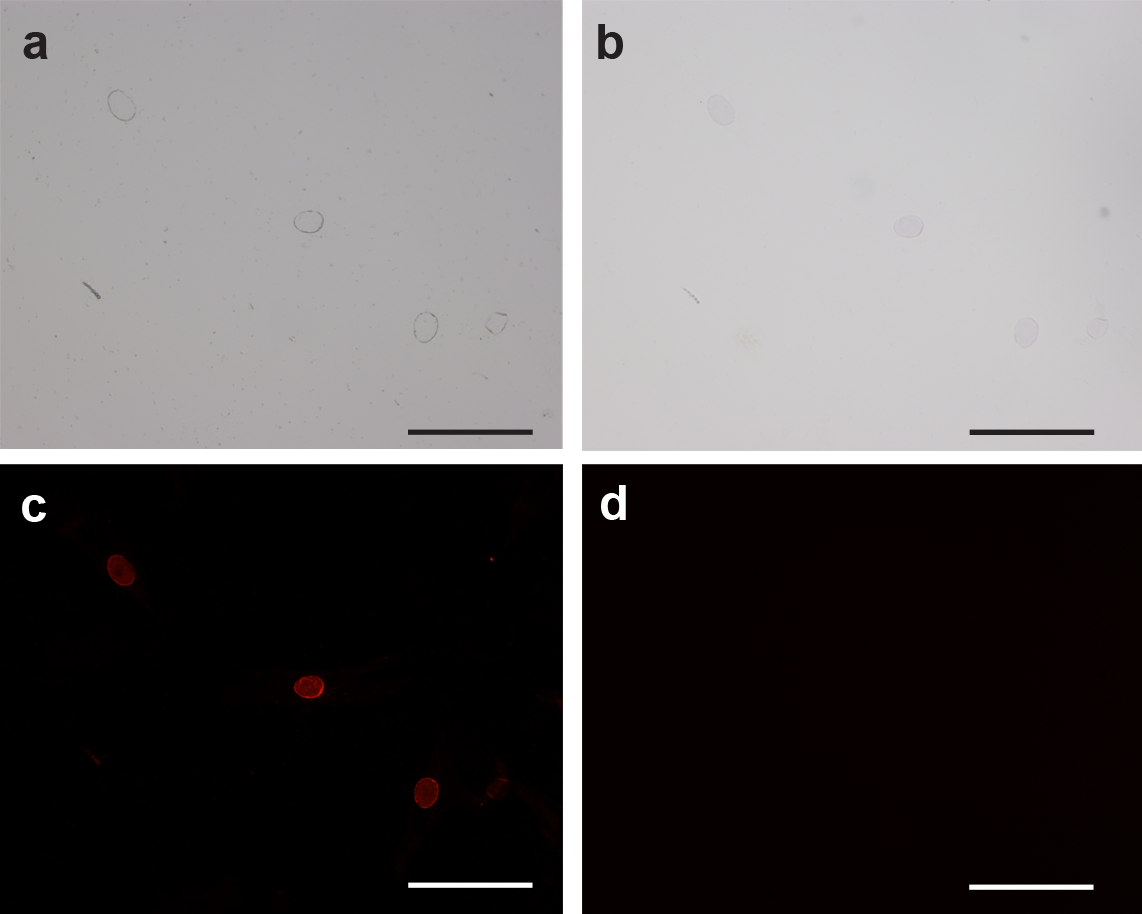

Supplement: S1 Fig — Quenching of fluorescent PBA by mounting medium. Human dermal fibroblasts were cultured on 8-well chamber slides, fixed, permeablized, blocked, labeled against nuclear pore complex, and polymerized in the presence of nile red fluorescent nanoparticles. The same representative frame imaged in brightfield (a) and in epifluorescent mode (c). After mounting with Vectashield hardset mounting medium, the same location was imaged in brightfield (b) and epifluorescent (d) imaging modes. Scale bars are 80 µm. (TIF) [file pone.0115630.s001.tif]

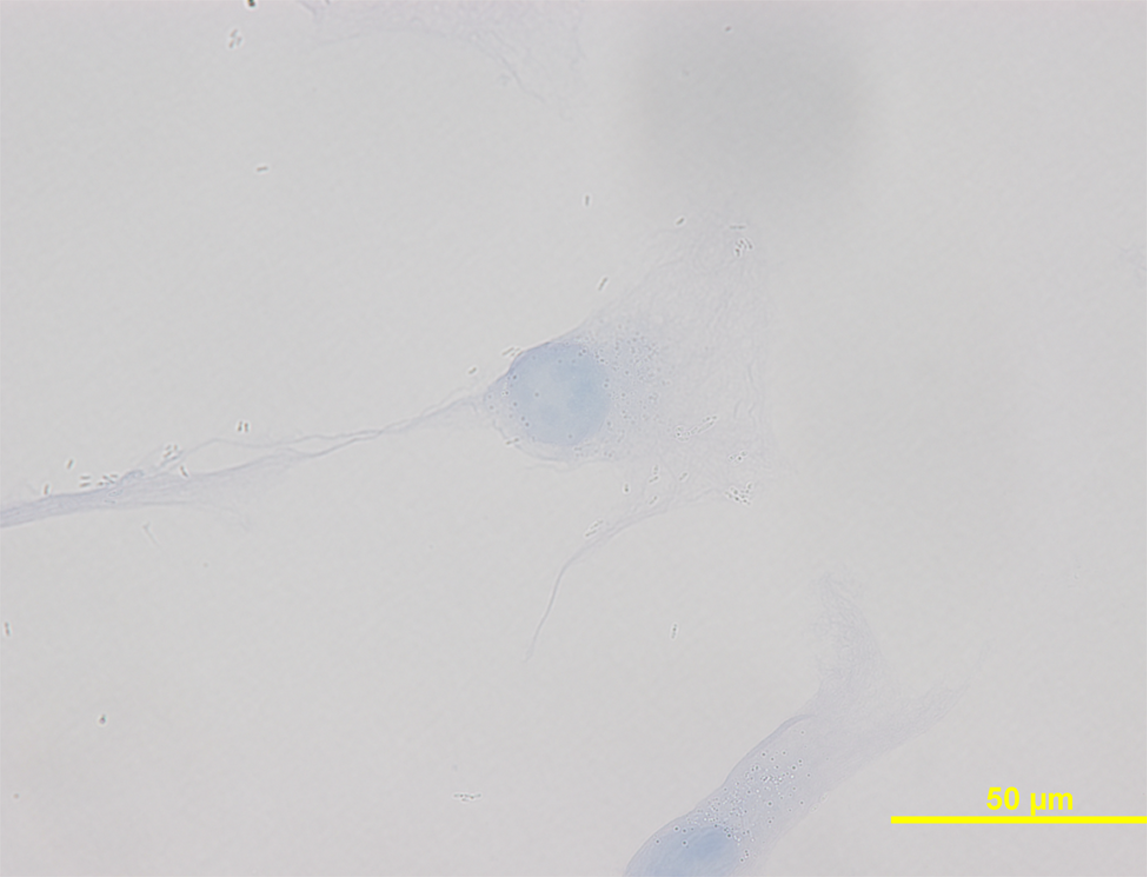

Supplement: S2 Fig — Control study showing limited nonspecific labeling of cells. Human dermal fibroblasts were cultured on 8-well chamber slides, fixed, permeabilized, blocked, and incubated in Evans Blue dye (1 mg/mL in PBS). (TIF) [file pone.0115630.s002.tif]
