# Supplementary material for: Interfacial Polymerization for Colorimetric Labeling of Protein Expression in Cells
Source: PLoS One. 2014 Dec 23;9(12):e115630. doi: 10.1371/journal.pone.0115630 (PMC4275217; doi:10.1371/journal.pone.0115630)
Supplement: S1 Table — Temporal staining intensity for Polymer Dye Labeling of nuclear pore complex. (DOCX) [file pone.0115630.s003.docx]

| Sample | Signal ^a,b^ | |
| --- | --- | --- |
|  | Mean | Standard Deviation |
| Polymer Dye Labeling day 0 | 0.363 | 0.088 |
| Polymer Dye Labeling day 208 | 0.343 | 0.091 |
| ^a^ - Signal is defined as the darkness of the nucleus.  ^b^ - Values are relative increase over empty region of slide. | | |
