# Supplementary material for: Interfacial Polymerization for Colorimetric Labeling of Protein Expression in Cells
Source: PLoS One. 2014 Dec 23;9(12):e115630. doi: 10.1371/journal.pone.0115630 (PMC4275217; doi:10.1371/journal.pone.0115630)
Supplement: S2 Table — Staining intensity for immunofluorescent labeling of nuclear pore complex. (DOCX) [file pone.0115630.s004.docx]

| Sample | Signal ^a,c^ | | Noise ^b,c^ | | Signal / Noise | |
| --- | --- | --- | --- | --- | --- | --- |
|  | Mean | Standard Deviation | Mean | Standard Deviation | Mean | Standard Deviation |
| Streptavidin-488 Labeling as in Figure 5a | 0.146 | 0.028 | 0.035 | 0.006 | 4.23 | 0.80 |
| Streptavidin-eosin Labeling as in Figure 5c | 0.101 | 0.008 | 0.022 | 0.007 | 4.53 | 0.36 |
| ^a^ - Signal is defined as the brightness of the nucleus.  ^b^ - Noise is defined as the brightness of the cytoplasm.  ^c^ - Values are relative increase over empty region of slide. | | | | | | |
